# Temperature Influences on Interactions Among Aflatoxigenic Species of *Aspergillus* Section *Flavi* During Maize Colonization

**DOI:** 10.3389/ffunb.2021.720276

**Published:** 2021-08-26

**Authors:** Connel Ching'anda, Joseph Atehnkeng, Ranajit Bandyopadhyay, Kenneth A. Callicott, Marc J. Orbach, Hillary L. Mehl, Peter J. Cotty

**Affiliations:** ^1^School of Plant Sciences, University of Arizona, Tucson, AZ, United States; ^2^International Institute of Tropical Agriculture (IITA), Lilongwe, Malawi; ^3^International Institute of Tropical Agriculture (IITA), Ibadan, Nigeria; ^4^United States Department of Agriculture - Agriculture Research Service, Tucson, AZ, United States; ^5^College of Food Science and Engineering, Ocean University of China, Qingdao, China

**Keywords:** *Aspergillus flavus*, *Aspergillus aflatoxiformans*, *Aspergillus parasiticus*, Lethal Aflatoxicosis Fungus, interspecific competition, aflatoxins

## Abstract

Fungal species within *Aspergillus* section *Flavi* contaminate food and feed with aflatoxins. These toxic fungal metabolites compromise human and animal health and disrupt trade. Genotypically and phenotypically diverse species co-infect crops, but temporal and spatial variation in frequencies of different lineages suggests that environmental factors such as temperature may influence structure of aflatoxin-producing fungal communities. Furthermore, though most species within *Aspergillus* section *Flavi* produce sclerotia, divergent sclerotial morphologies (small or S-type sclerotia vs. large or L-type sclerotia) and differences in types and quantities of aflatoxins produced suggest lineages are adapted to different life strategies. Temperature is a key parameter influencing pre- and post-harvest aflatoxin contamination of crops. We tested the hypothesis that species of aflatoxin-producing fungi that differ in sclerotial morphology will vary in competitive ability and that outcomes of competition and aflatoxin production will be modulated by temperature. Paired competition experiments between highly aflatoxigenic S-type species (*A. aflatoxiformans* and Lethal Aflatoxicosis Fungus) and L-type species (*A. flavus* L morphotype and *A. parasiticus*) were conducted on maize kernels at 25 and 30°C. Proportions of each isolate growing within and sporulating on kernels were measured using quantitative pyrosequencing. At 30°C, S-type fungi were more effective at host colonization compared to L-type isolates. Total aflatoxins and the proportion of B vs. G aflatoxins were greater at 30°C compared to 25°C. Sporulation by L-type isolates was reduced during competition with S-type fungi at 30°C, while relative quantities of conidia produced by S-type species either increased or did not change during competition. Results indicate that both species interactions and temperature can shape population structure of *Aspergillus* section *Flavi*, with warmer temperatures favoring growth and dispersal of highly toxigenic species with S-type sclerotia.

## Introduction

Aflatoxins are carcinogenic secondary metabolites produced by several species within *Aspergillus* section *Flavi*. Aflatoxins contaminate food and feed worldwide and are a health concern, causing liver cancer, stunting of growth, and immune suppression at lower concentrations and rapid death after very high exposure (Gong et al., 2004; Williams et al., 2004; Liu and Wu, 2010). Aflatoxins are also an economic burden to farmers, traders, and nations due to loss of revenue as a result of strict regulations that limit sale of contaminated crops (Mitchell et al., 2016). It is estimated that strict regulations of aflatoxins by the European Union cost African maize exporters over $670 million annually (Wu, 2015). Though aflatoxin contamination is a perennial problem in some regions, the frequency and severity of aflatoxin contamination events is highly variable and dependent on factors including compositions of fungal communities associated with crops and environmental conditions such as temperature (Cotty et al., 2008; Probst et al., 2010).

Aflatoxin-producing fungi within *Aspergillus* section *Flavi* are phenotypically and genotypically diverse with large inter- and intra-specific variation in quantities of asexual spores (conidia) produced, size and quantities of sclerotia produced, and types and quantities of aflatoxins produced in crops (Cotty et al., 1994; Mehl and Cotty, 2010; Frisvad et al., 2019). *Aspergillus* section *Flavi*, species produce sclerotia that are either large (L-type > 400 μm) or small (S-type <400 μm). Most lineages within *Aspergillus* section *Flavi* demonstrate a single sclerotial type, while *A. flavus*, the most common causal agent of aflatoxin contamination, includes both S and L sclerotial types (Cotty, 1989). There are several phylogenetically distinct species within *Aspergillus* section *Flavi*, some of which were previously misidentified as *A. flav*us S-type, that have the S-type morphology. These species include *A. texensis* (Singh et al., 2018), *A. toxicus* (Singh et al., 2020)*, A. agricola* (Singh et al., 2020), *A. aflatoxiformans* (Cotty and Cardwell, 1999; Frisvad et al., 2019), and the unnamed Lethal Aflatoxicosis Fungus (LAF), a species closely related to *A. minisclerotigenes* and associated with multiple deaths in Kenya in 2004 (Probst et al., 2007, 2012; Frisvad et al., 2019). *Aspergillus parasiticus* is another primary causal agent of aflatoxin contamination (Horn, 2003; Klich, 2007), and though it can be distinguished morphologically from *A. flavus* based on colony color and conidial ornamentation, it produces L-type sclerotia (Frisvad et al., 2019). On certain culture media, S-type species tend to produce fewer conidia and abundant sclerotia whereas L-type species produce copious amounts of conidia and few sclerotia (Cotty, 1989).

In addition to variation in morphological characteristics, species within *Aspergillus* section *Flavi* also vary in the types and quantities of aflatoxins produced. *Aspergillus parasiticus* and *A. aflatoxiformans* produce both B and G aflatoxins, while *A. flavus* and LAF produce only B aflatoxins. Among the four types of aflatoxins, aflatoxin B_1_ is the most potent carcinogen followed by aflatoxins G_1_, B_2_ and G_2_ respectively (Hernandez-Martinez and Navarro-Blasco, 2010; Liu and Wu, 2010). Furthermore, the S-type fungi and *A. parasiticus* consistently produce high levels of aflatoxins, while aflatoxin production by *A. flavus* L-type is highly variable (Cotty, 1989; Probst et al., 2007). Interactions among these species that differ in morphology and aflatoxin production have important implications for the etiology of crop aflatoxin contamination in regions where these species co-occur (Nesci and Etcheverry, 2002; Barros et al., 2005; Giorni et al., 2007; Atehnkeng et al., 2008a; Donner et al., 2009; Probst et al., 2010; Diedhiou et al., 2011; Kachapulula et al., 2017; Agbetiameh et al., 2018; Singh and Cotty, 2019; Sserumaga et al., 2020). In some parts of sub-Saharan Africa, *A. flavus* L-type, *A. parasiticus, A. aflatoxiformans* and LAF are the most frequently co-occurring species. Temporal and spatial co-occurrence of *Aspergillus* species can lead to interspecific competition for nutrients, space, and other limiting resources, with potential impacts on the quantity and type of aflatoxins produced in crops.

Some studies suggest differential niche adaptation within lineages of *Aspergillus* section *Flavi*. For example, based on morphological and genomic features it can be inferred that S-type and L-type species vary in their life strategies in the environment and during crop colonization (Cotty et al., 1994; Ohkura et al., 2018). Abundant sporulation by L-type fungi may provide a dispersal advantage in the phyllosphere, allowing for exploitation of new nutrient environments or hosts, while allocating more resources to production of mycelia may confer an advantage in invading host tissues and acquiring nutrients (Cotty et al., 1994; Mehl and Cotty, 2010; Mehl et al., 2012). Abundant production of sclerotia may be advantageous for survival in the soil environment (Garber and Cotty, 1997; Mehl et al., 2012; Ohkura et al., 2018) where microbial competition is high. This contrasts with the phyllosphere where microbial species diversity is lower (Lindow and Brandl, 2003; Delmotte et al., 2009) and interacting fungi may use different strategies to compete. Some genotypes of *A. flavus* are highly competitive during host colonization, while others are less competitive in terms of invasion and nutrient acquisition but outcompete other fungi through greater dispersal of conidia (Mehl and Cotty, 2010; Sweany et al., 2011). While intraspecific interactions among *A. flavus* genotypes in different nutrient environments and hosts has been studied (Mehl and Cotty, 2013a,b), little is known about interspecific interactions between different *Aspergillus* section *Flavi* species.

In addition to adaptation to hosts or nutrient environments, there is also evidence that species in *Aspergillus* section *Flavi* may be adapted to different abiotic conditions such as temperature. For example, *A. parasiticus* colonizes crops at lower temperatures than *A. flavus* and grows faster than *A. flavus* in culture media at temperatures between 22 and 25°C; however, it grows slower than *A. flavus* at temperatures above 30°C (Pitt and Miscamble, 1995; Horn, 2005). The S-type fungi are more frequently reported (up to 80%), in the tropics, subtropics and desert environments where average temperatures are high (>25^o^C), suggesting that the S-type fungi are adapted to these environments (Cardwell and Cotty, 2002; Pildain et al., 2004, 2008; Donner et al., 2009; Perrone et al., 2014). Temperature also plays a crucial role in sporulation and aflatoxin production. Higher temperatures favor growth, sporulation, and dispersal of *A. flavus* (Payne et al., 1985; Diener et al., 1987; Scheidegger and Payne, 2003; Jaime-Garcia and Cotty, 2010). At temperatures below 20°C, *Aspergillus* section *Flavi* species occur in low frequencies while at temperatures >25°C, aflatoxin-producing fungi are common throughout the soil, the air, and on crop surfaces (Manabe et al., 1978; Shearer et al., 1992; Cotty and Jaime-Garcia, 2007). High levels of aflatoxin production have been observed between 25 and 35°C (Schmidt-Heydt et al., 2009), with aflatoxin production by *A. flavus* often being maximal at 30°C (Bhatnagar et al., 2006; O'Brian et al., 2007).

Since genetically and phenotypically diverse species of *Aspergillus* section *Flavi* coexist in agricultural environments, interspecies competition is likely to impact the composition and aflatoxin-producing potential of crop-associated fungal communities. Abiotic factors including temperature will likely influence both competition among species (Bock et al., 2004; Hiscox et al., 2016) and the extent to which aflatoxin biosynthesis occurs (Singh et al., 2020). We test the hypothesis that temperature will impact both competition between L-type and S-type species of *Aspergillus* section *Flavi* and the dynamics of aflatoxin production. The objectives of the current study were to: (1) quantify outcomes of competition between *Aspergillus* species differing in sclerotial type and aflatoxin production (*A. flavus, A. parasiticus, A. aflatoxiformans*, and LAF) and (2) evaluate the impacts of temperature on interspecific interactions and aflatoxin production during colonization of maize kernels.

## Materials and Methods

### Fungal Isolates

Four highly aflatoxigenic isolates were used in this study: two L-type, *A. flavus* (AF13) and *A. parasiticus* (AP2999), and two S-type, *A. aflatoxiformans* (BN008-R) and LAF (K0550K). Information for these isolates including the types of aflatoxins they produce are listed in Table 1. Cultures started from single conidia were cultivated on 5/2 agar (5% V8 juice, 2% agar, pH 6.0) (Cotty, 1989), and plugs from growing colonies were transferred into 4 ml of sterile water for storage. Conidial suspensions were prepared from 7-day-old cultures grown on 5/2 agar as described previously (Mehl and Cotty, 2013a). Concentrations of conidia were quantified using a turbidity meter (Turbidimeter TB 300IR; Orbeco Analytical Systems, Farmingdale, NY), and conidia per ml were calculated with a nephelometric turbidity unit (NTU) vs. CFU standard curve (conidia per ml = 49,937 x NTU) (Bock and Cotty, 1999; Probst et al., 2010).

**Table 1:** *Aspergillus* isolates used in the current study.

**Isolate**	**Species**	**Source**	**Sclerotial type^**a**^**	**Aflatoxin production^**b**^**	**References**
AF13	*A. flavus*	Soil, USA	L	B	Cotty, 1989
AP2999	*A. parasiticus*	Peanut, Uganda	L	B, G	Rambo et al., 1974
BN008-R	*A. aflatoxiformans*	Soil, Benin	S	B, G	Cotty and Cardwell, 1999
K0550-K	LAF^c^	Maize, Kenya	S	B	Probst et al., 2012

a *S, small sclerotia; L, large sclerotia.*

b *B, B aflatoxins; G, G aflatoxins.*

c *LAF, Lethal Aflatoxicosis Fungus, an unnamed taxon closely related to A. minisclerotigenes*.

### Competition Experiments

Competition experiments were conducted on mature maize kernels (Pioneer hybrid N82VGT) that were sterilized by autoclaving (121°C, 20 min) (Probst and Cotty, 2012). Maize kernel inoculations were conducted as described previously (Mehl and Cotty, 2013a) with some modifications. Maize kernel moisture content was measured using a moisture analyzer (HB43 Halogen Moisture Analyzer, Mettler-Toledo), and the final moisture content of the maize was adjusted to 30% with either sterile water or sterile water plus the inoculum. The four isolates were inoculated singly or paired in all possible combinations, resulting in four single isolate treatments and six co-inoculation treatments. In the treatments, 5 g sterile maize in 250-ml Erlenmeyer flasks sealed with gas-permeable BugStopper plugs were inoculated with either 5 × 10^4^ conidia of one isolate or with suspensions that contained 5 × 10^4^ conidia of each isolate (10^5^ conidia total) for co-inoculation treatments.

A total of eight flasks per treatment were prepared: four for aflatoxin analyses and four for conidia quantification and DNA analyses. Eight flasks of uninoculated maize were included as controls. The treatment and control flasks were incubated for seven days in the dark at either 25 or 30°C, temperatures that are common in agricultural environments where maize is grown and where *Aspergillus* species and aflatoxin contamination are common. At the end of the incubation period, maize kernels were washed with 20 ml 0.01% Tween-80 followed by 20 ml of distilled water to recover conidia. Washings were sieved using Miracloth (EMD Millipore, Billerica, MA) to separate sclerotia from the conidial suspension, transferred into 50 ml conical tubes, and measured for turbidity. The quantities of conidia were calculated from turbidity as described above.

Following centrifugation of the conidial suspensions (4400 g, 5 min), DNA was extracted from the conidial pellet using a previously described protocol (Callicott and Cotty, 2015). Washed kernels were immediately dried at 60^o^C for 48 h and then ground for 15 s in an analytical mill (IKA Works, Wilmington, NC) for DNA extraction representing the colonizing mycelial DNA. Colonizing mycelial DNA was extracted from 200 mg of ground maize kernels by modifying the method described for conidial DNA extraction. In short, 800 μl lysis buffer was added to 200 mg of ground kernels as starting material, and all subsequent steps were followed as described previously (Callicott and Cotty, 2015).

### Pyrosequencing Assays

Sequences were obtained from GenBank for the nitrate reductase (*niaD*), aflatoxin transcription factor (*aflR*) and calmodulin (*cmdA*) genes of *A. flavus* (AF13 GenBank accessions MH760530, MH752568, and MK119698), *A. parasiticus* (AP2999 GenBank accessions MH76053, KT829482, and MK119703) and *A. aflatoxiformans* (BN008-R GenBank accessions MK119681, AF441441, and MK119715). The sequences for *aflR, cmdA, and niaD* of the LAF isolate (K0550K GenBank accessions MZ673642, MZ673640 and MZ673641) were determined using PCR amplification and bidirectional sequencing using the primers and conditions described previously (Probst et al., 2012; Singh and Cotty, 2019). Sequences were aligned using MUSCLE (Edgar, 2004) within Geneious Pro Version 7.1.9 (Biomatters Ltd, Auckland, New Zealand). Putative single nucleotide polymorphisms (SNPs) were identified visually from the alignment and were confirmed by pyrosequencing. Species-specific pyrosequencing assays based on these SNPs were designed using PyroMark Assay Design software v2.0.1.15 (Qiagen, Germantown, MD). PCR primer pairs 5′-Biotin-GGTTTTGGATCTGACCAGTGTAG-3′ / 5′-GGCAGATAGTACCCGGCTTG-3′ and a sequencing primer 5′-TAGTACCCGGCTTGC-3′ targeted an *A. flavus-*specific SNP in the *aflR* gene. PCR primer pairs 5′-Biotin-CGGGCTGGCCATTTATTATGAT-3′ / 5′-GGGAAGACGGGCGTTGTTTA-3′ and sequencing primer 5′-GGAACCGACCCGACT-3′ targeted an *A. aflatoxiformans*-specific SNP in the *cmdA* gene region. PCR primer pairs 5′-Biotin-GGCTGAAGAGGCTGATCTTGAC-3′ / 5′-CGCGGTTGTCATTGATATGGTA-3′ and sequencing primer 5′-GTCATTGATATGGTACCAG-3' targeted an *A. parasiticus*-specific SNP in the *niaD* gene region. These three sets of primers distinguished between LAF and the species being targeted by the pyrosequencing assay. PCR reactions were performed in Bioneer AccuPower Hotstart PCR PreMix tubes (Bioneer, Inc., Alameda, CA). For each primer pair, one primer was 5′ biotinylated and HPLC-purified. The reactions were performed in 20 μl and included 0.25 μM each primer and 10 ng genomic DNA. PCR conditions for all the assays were: DNA denaturation at 94°C for 5 min, followed by 38 cycles of denaturation at 94°C for 20 s, primer annealing at 62°C for 30 s, extension at 72°C for 30 s, and a final extension step at 72°C for 5 min. Amplicons were visualized with GelRed on 1.0% agarose gels before pyrosequencing to confirm amplification and correct amplicon size for each assay. Proportions of species-specific SNPs in pools of amplicons were quantified by pyrosequencing using a PyroMark Q48 Autoprep Pyrosequencer (Qiagen, Germantown MD), following the manufacturer's instructions. Percentages of DNA from each isolate were deduced from the proportions of species-specific SNPs calculated using PyroMark Q48 Autoprep software v.2.4.2. No amplicons were detected from uninoculated control maize.

### Aflatoxin Extraction and Quantification

Aflatoxins were extracted from maize kernels following inoculation and incubation by adding 50 ml of 70% methanol to each flask and homogenizing the kernels/methanol using a laboratory grade blender (Waring Laboratory, Torrington, CT, USA) at full speed for 30 s. Extracts were separated by thin layer chromatography (TLC) (Silica gel 60 plates, EMD, Darmstadt, Germany) in ethyl ether-methanol-water (96:3:1) along with an aflatoxin standard (Aflatoxin Mix Kit-M, Supelco, Bellefonte, PA, USA). Aflatoxins on TLC plates were visualized using 365-nm UV light and quantified directly with a scanning densitometer (TLC Scanner 3; Camag Scientific Inc, Wilmington, NC, USA) (Pons et al., 1969). Peaks generated by a scanning densitometer were used to estimate aflatoxin concentrations by comparing the area under the peak generated by sample to the area under the peak generated by a standard. There were no detectable aflatoxins in uninoculated control flasks. The limit of quantification was 10 μg/Kg.

### Experimental Design and Data Analysis

The experiment was a randomized factorial design (ten inoculation treatments by two temperatures) with four replicates (one flask per replicate). Data were analyzed in JMP 11.1.1 (SAS Institute, Cary, NC, USA, 2013). Conidial quantities and aflatoxin data were log-transformed, and isolate percentages were arcsine transformed before analysis. Percentages of coinfecting isolates were compared using Student's *t*-test. The influence of temperature and inoculation treatment on sporulation and aflatoxin production was analyzed using a factorial analysis of variance. Mean separation was done using Tukey's HSD. Expected percentages of conidia for each isolate were calculated based on quantities of conidia produced from individual inoculations using the formula:


$$\begin{array}{l} \%X_{e}\;=100\times (X_{c})\;/\;(X_{c}\;+\;Y_{c}) \end{array}$$

where *X*_*e*_= expected percent isolate, *X*_*c*_ = quantities of conidia produced by isolate “X” grown individually, and *Y*_*c*_ = quantities of conidia produced by isolate “Y” grown individually.

Conidia produced by each species during competition were calculated by multiplying the total number of conidia per gram host substrate by the proportion of each isolate in the conidia DNA using the formula:


$$\begin{array}{l} \;M\;=\;C\;\times \;K \end{array}$$

where M = calculated isolate M conidia, C = total conidia, K = proportion of isolate M from conidia DNA.

Proportion of aflatoxins comprised of B aflatoxins was calculated using the formula:


$$\begin{array}{l} \%B=100\times (T_{B})\;/\;(T_{B}\;+\;T_{G})\; \end{array}$$

where %B = proportion of aflatoxins comprised of B aflatoxins, T_B_ = total B aflatoxins, T_G_ = total G aflatoxins. Expected total aflatoxin production by co-inoculated isolates was calculated using the formula:


$$\begin{array}{l} T_{e}\;=(T_{m}\;\times \;\%M_{m})+\;(T_{n}\;\times \;\%N_{m}) \end{array}$$

where T_e_ = expected total aflatoxin, T_m_ = aflatoxin produced by isolate “M” grown individually, T_n_ = aflatoxin produced by isolate “N” grown individually, %M_m_ = % isolate “M” in mycelia, %N_m_ = % isolate “N” in mycelia.

The expected and observed aflatoxins or expected and measured conidial percentages were compared using paired *t*-test. Significant differences are reported at α = 0.05. Non-transformed means are reported for clarity.

## Results

### Influence of Temperature on Interactions Among Species During Maize Kernel Colonization

**Figure 1:**
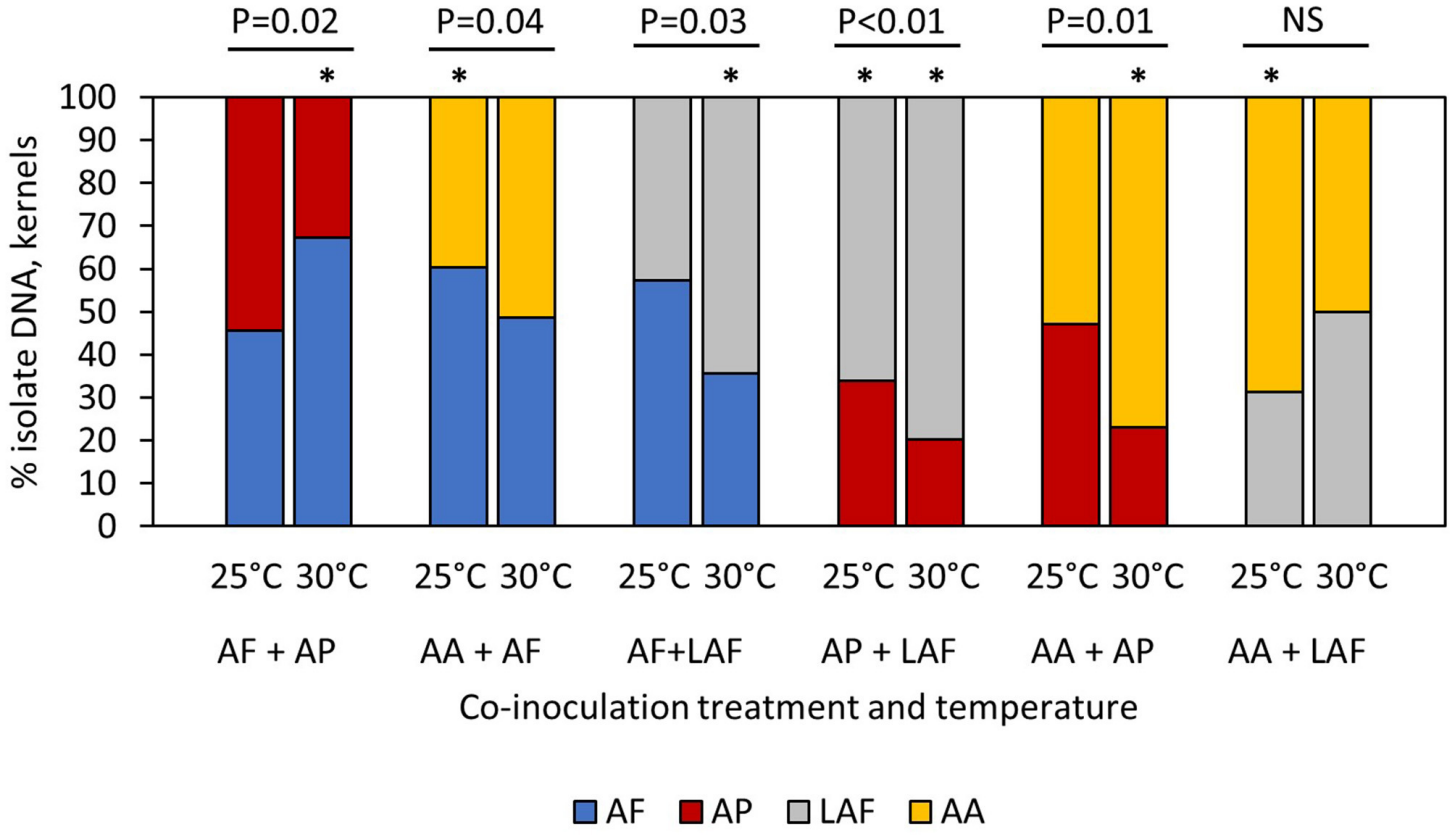
Influence of temperature on competition between co-inoculated *Aspergillus* isolates during maize kernel colonization. AF, *A. flavus* L strain (AF13) (L-type); AP, *A. parasiticus* (AP2999) (L-type); AA, *A. aflatoxiformans* (BN008-R) (S-type); LAF, Lethal Aflatoxicosis Fungus (K0550K) (S-type). Asterisks above individual bars indicate treatments with percentages that are significantly different than 50% (*t*-test, *P* < 0.05). P values above the groups of paired bars indicate outcomes of competition were significantly different at 25 and 30°C (*t*-test, *P* < 0.05). NS indicate not significant.

When four different *Aspergillus* species were co-inoculated on maize kernels, relative colonization of the species was temperature and isolate dependent, as measured by the proportion of isolate-specific mycelial DNA in the kernels (Figure 1). The proportion of *A. flavus* colonizing kernels was not influenced by temperature (*P* = 0.5299) or the identity of the co-infecting isolate (*P* = 0.3901), but there was a significant temperature by co-inoculated isolate interaction (*P* = 0.0246). For example, proportions of *A. flavus* and *A. parasiticus* were equal at 25°C, but the proportion of *A. flavus* was significantly greater than *A. parasiticus* at 30°C (Figure 1). In contrast, the proportion of *A. flavus* was greater than *A. aflatoxiformans* at 25°C, but the two isolates were equal at 30°C. When co-inoculated with other isolates, the proportion of *A. parasiticus* was significantly greater at 25°C compared to 30°C (*P* < 0.0001*)*, but proportions of *A. parasiticus* DNA were not influenced by the co-infecting isolate (*P* = 0.4015) or the interaction between temperature and co-infecting isolate (*P* = 0.1917). When co-inoculated with other isolates, the proportion of LAF was influenced by the main factors of co-inoculated isolate (*P* = 0.0409) and temperature (*P* < 0.0001) but there were no interactions among factors (*P* = 0.0772). Proportions of LAF DNA in kernels, indicating colonization, were high at 30°C compared to 25°C, and overall, the proportion of LAF was greater than the two L-type fungi (*A. flavus* and *A. parasiticus*) but was equal to the other S-type isolate (*A. aflatoxiformans*). Overall, the proportions of *A. aflatoxiformans* DNA in kernels did not differ between 25 and 30°C, but proportions were influenced by the co-inoculated isolate (*P* = 0.0013) and the interaction between co-inoculated isolate and temperature (*P* = 0.0005). When co-inoculated with *A*. *parasiticus* or *A. flavus*, proportions of *A. aflatoxiformans* were greater at 30°C compared to 25°C. However, when co-inoculated with LAF, proportions of *A. aflatoxiformans* DNA in kernels were lower at 30°C compared to 25°C (Figure 1).

### Influence of Temperature and Co-inoculation on Aflatoxin Production

When aflatoxin production was measured following co-inoculation of the four *Aspergillus* species on maize, total aflatoxin production varied by both inoculation treatment (*P* < 0.0001) and temperature (*P* < 0.0001), and there was an interaction between the two factors (*P* < 0.0001). Individual and paired isolates produced similar quantities of total aflatoxins at 25°C. Except for *A. flavus* alone or when it was co-inoculated with LAF, greater concentrations of total aflatoxins were produced at 30°C compared to 25°C (Table 2). Among the individually inoculated isolates, *A. parasiticus* produced the greatest concentrations of aflatoxins and *A. flavus* produced the least at 30°C. For maize co-inoculated with either *A. flavus* and *A. parasiticus* or *A. aflatoxiformans* and LAF, aflatoxin concentrations at 30°C were similar to what would be expected based on the aflatoxin-producing potential of individual isolates and the proportions of each isolate colonizing the kernels. However, measured aflatoxin concentrations were greater than expected in kernels co-inoculated with *A*. *aflatoxiformans* and either *A. flavus* (354 vs. 184 μg/g; *P* = 0.0007) or *A. parasiticus* (455 vs. 311 μg/g; *P* = 0.0394). In contrast, measured aflatoxin concentrations were less than expected for kernels co-inoculated with LAF and either *A. flavus* (60 vs. 133 μg/g; *P* < 0.0001) or *A. parasiticus* (175 vs. 235 μg/g; *P* = 0.0262).

**Table 2:** Effect of temperature and co-inoculation of *Aspergillus* isolates on aflatoxin production on maize kernels at 25 and 30°C.

**Inoculation treatment^**x**^**	**Total aflatoxin (μg/g)** ^**y**^			**% B aflatoxin** ^**y**^	
	**25^**°**^C**	**30^**°**^C**	**Fold change^**z**^**	**25^**°**^C**	**30^**°**^C**
AF	54 a	85 f	1.6	100 a	100 a
AP	88 a	414 ab*	4.7	26 d	62d e*
LAF	61 a	158 ef*	2.6	100 a	100 a
AA	64 a	278 cde*	4.3	24 d	63 de*
AF + AP	66 a	228 de*	3.5	72 b	82 b
AA + AF	94 a	354 abc*	3.8	64 bc	71 c
AF + LAF	81 a	60 f	0.7	100 a	100 a
AP + LAF	62 a	175 ef*	2.8	60 c	83 b*
AA + AP	53 a	455 a*	8.6	30 d	59 e*
AA + LAF	48 a	307 bdc*	6.4	56 c	64 c

*^x^AF, A. flavus L strain (AF13) (L-type); AP, A. parasiticus (AP2999) (L-type); AA, A. aflatoxiformans (BN008-R) (S-type); LAF, Lethal Aflatoxicosis Fungus (K0550K) (S-type).*

*^y^Total aflatoxins were quantified from TLC plates using a scanning densitometer. Proportion of B aflatoxins was calculated: %B = 100 × (T_B_)/(T_B_ + T_G_), where %B = proportion of aflatoxins comprised of B aflatoxins, T_B_ = total B aflatoxins, T_G_ = total G aflatoxins. Means followed by the same letter within a column are not significantly different (P >0.05) according to Tukey's HSD. Asterisk denotes significant difference between 25 and 30°C treatments (t-test, P < 0.05).*

*^z^Fold change was calculated by dividing the total aflatoxins produced at 30°C by the total aflatoxins produced at 25°C*.

Two of the isolates, *A. parasiticus* and *A. aflatoxiformans*, produced both B and G aflatoxins, and the proportion of total aflatoxins comprised of B aflatoxin varied by inoculation treatment (*P* < 0.0001), temperature (*P* < 0.0001), and the interaction of the two factors (*P* < 0.0001). At 25°C, B aflatoxins comprised 30% or less of the total aflatoxins produced by *A. parasiticus* and *A. aflatoxiformans* grown individually or together, but at 30°C, over 50% of aflatoxins produced were B aflatoxins (Table 2).

### Influence of Temperature on Interactions Among Species Sporulating on Maize Kernels

Similar to the results for colonization, the interaction between temperature and co-inoculated isolate influenced the proportions of different species sporulating on maize kernels (*P* < 0.0001, Table 3). In general, proportions of isolates colonizing and sporulating on kernels were similar. However, the proportion of *A. flavus* was greater during sporulation compared to colonization when co-inoculated with either *A. parasiticus* at 25°C (*P* = 0.0034) or *A. aflatoxiformans* at 30°C (*P* = 0.0010). Comparisons between expected proportions of conidia produced by each isolate during co-infection (calculated based on sporulation of individually inoculated isolates) and measured proportions are summarized in Table 3. The proportion of *A. flavus* was greater than expected when co-inoculated with *A. parasiticus* at both temperatures (25°C: *P* = 0.0493; 30°C; *P* = 0.0023). However, the proportion of *A. flavus* was less than expected when co-inoculated with *A. aflatoxiformans* at 25°C (*P* = 0.0009) and 30°C (*P* = 0.0213) and with LAF at 30°C (*P* = 0.0082). Proportions of *A. parasiticus* were less than expected when co-inoculated with *A. flavus* (*P* = 0.0071) and *A. aflatoxiformans* (*P* = 0.0026) at 25°C and when co-inoculated with any other species at 30°C (Table 3). In contrast, the proportion of *A. aflatoxiformans* was greater than expected (*P* < 0.0001) at 25°C when co-inoculated with LAF. However, the proportions of the two species were equal (*P* = 0.0529) at 30°C (Table 3).

**Table 3:** Influence of temperature on predicted and measured conidia percentages from paired co-inoculation of *Aspergillus* isolates at 25 and 30°C.

**Measured isolate**	**Co-inoculated isolate^**v**^**	**Percent isolate DNA, conidia** ^**u**^				**25 vs 30**^**°**^**C**, ***P*** **value**^**z**^	
		**25** ^**°**^ **C**		**30** ^**°**^ **C**			
		**Expected^**w**^**	**Measured^**x**^**	**Expected**	**Measured**	**Expected**	**Measured**
AF	AP	51 b	63 a*	60 b	81 a*	0.1413	0.0096
	AA	84 a	63 a*	92 a	77 a*	0.0273	0.0138
	LAF	64 ab	62 a	95 a	54 b*	0.0006	0.4139
	*P* value^y^	0.0004	0.9601	<0.0001	0.0083		
AP	AF	49 b	37 b*	40 b	19 a*	0.1593	0.0084
	AA	84 a	41 b*	87 a	25 a*	0.3713	0.0419
	LAF	63 b	63 a	92 a	30 a*	0.0013	<0.0001
	*P* value^y^	0.0003	0.001	<0.0001	0.0608		
AA	AF	16 b	37 b*	8 b	23 c*	0.0272	0.0089
	AP	16 b	59 a*	13 b	75 a*	0.425	0.0451
	LAF	25 a	67 a*	62 a	50 b	0.0001	0.0077
	*P* value^y^	0.0285	0.0015	<0.0001	<0.0001		
LAF	AF	36 b	38 a	5 b	46 b*	0.002	0.4162
	AP	37 b	37 a	8 b	70 a*	0.0033	<0.0001
	AA	75 a	33 a*	38 a	50 b	0.0001	0.0092
	*P* value^y^	0.0002	0.6243	<0.0001	0.0064		

*^u^Percentages followed by the same letter are not significantly different according to Tukey's HSD (P > 0.05) (n = 4). Asterisks denote significant differences between measured and expected percentages using t test (P < 0.05).*

*^v^AF, A. flavus L strain (AF13) (L-type); AP, A. parasiticus (AP2999) (L-type); AA, A. aflatoxiformans (BN008-R) (S-type); LAF, Lethal Aflatoxicosis Fungus (K0550K) (S-type).*

*^w^Expected conidia percentages were calculated based on conidia quantities produced from individual inoculations: %X_e_ = 100 × (X_c_)/(X_c_ + Y_c_), where X_e_= expected percent isolate, X_c_ = quantities of conidia produced by isolate “X” grown individually, and Y_c_ = quantities of conidia produced by isolate “Y” grown individually. ^x^Measured percentages of each isolate were quantified by quantitative pyrosequencing of isolate-specific SNPs from total conidial DNA (n = 4).*

*^y^Isolate percentages were compared by Tukey's HSD (n = 4).*

*^z^Expected or measured conidia percentages of each isolate at 25 and 30°C were compared using t-test (n = 4)*.

**Figure 2:**
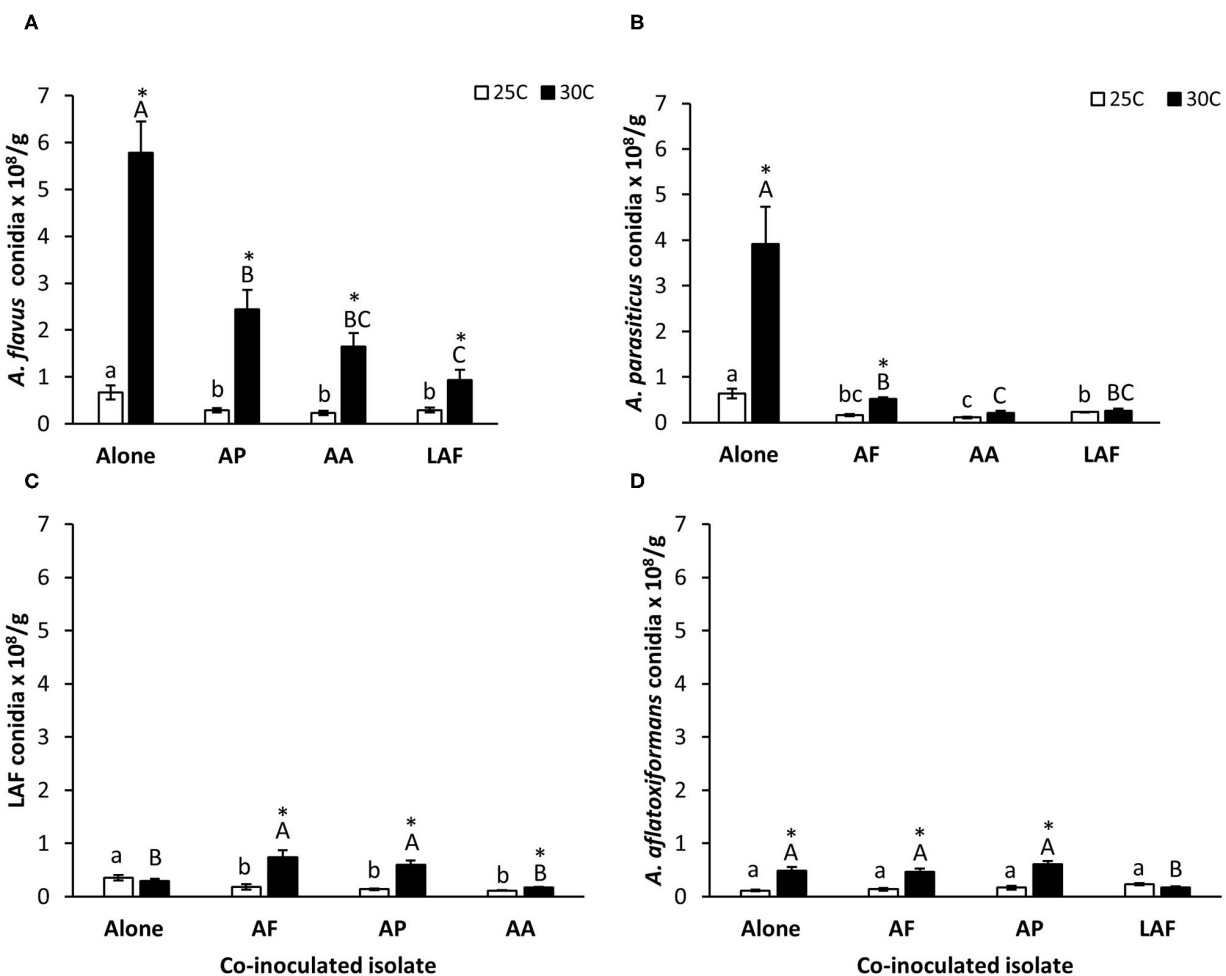
Effect of temperature and co-inoculation of *Aspergillus* isolates on sporulation during growth on maize kernels at 25 and 30°C, **(A)** *A. flavus* conidia alone and in competition with other isolates, **(B)** *A. parasiticus* conidia alone and in competition with other isolates, **(C)** LAF conidia alone and in competition with other isolates and **(D)** *A. aflatoxiformans* conidia alone and in competition with other isolates. AF, *A. flavus* L strain (AF13) (L-type); AP, *A. parasiticus* (AP2999) (L-type); AA, *A. aflatoxiformans* (BN008-R) (S-type); LAF; Lethal Aflatoxicosis Fungus (K0550K) (S-type). Quantities of conidia produced by each species during competition were determined by multiplying the proportion of conidial DNA of each isolate measured using quantitative pyrosequencing by the total number of conidia produced by the co-inoculated isolates. Bars followed by similar letters (lower case letters 25°C treatment and uppercase letters 30°C treatment) are not significantly different (*P* > 0.05) Tukey's HSD. Asterisks denote significant difference between 25 and 30°C treatments (*t*-test, *P* < 0.05).

Quantities of conidia produced by each isolate growing individually or in competition with another isolate on maize kernels are summarized in Figure 2. Except for LAF, sporulation by the individually inoculated isolates was greater at 30°C than 25°C. The impact of co-inoculation on sporulation varied by isolate and temperature. Sporulation by *A. flavus, A. parasiticus*, and LAF was influenced by co-inoculated isolate (*P* < 0.0001, *P* < 0.0001, and *P* < 0.0001 respectively), temperature (*P* < 0.0001, *P* < 0.0001, and *P* < 0.0001, respectively) and the interaction of the two factors (*P* = 0.0372, *P* < 0.0001, and *P* < 0.0001, respectively). In contrast, sporulation by *A. aflatoxiformans* was dependent on temperature (*P* < 0.0001) and the interaction between temperature and co-inoculated isolate (*P* < 0.0001) but not by the identity of the co-inoculated isolate (*P* = 0.0735). Co-inoculation suppressed sporulation (*P* < 0.05) by *A. flavus* (Figure 2A) and *A. parasiticu*s (Figure 2B) at both 25°C and 30°C. However, the effects of co-inoculation on sporulation by the S morphology species, *A. aflatoxiformans* and LAF, was more variable. Co-inoculation reduced sporulation by LAF at 25°C, but at 30°C LAF produced more conidia during co-inoculation with *A. flavus* and *A. parasiticus* compared to when it grew alone (Figure 2C). Sporulation by *A. aflatoxiformans* was the least affected during co-inoculation at both temperatures, with reductions only occurring at 30°C during co-inoculation with LAF (Figure 2D).

## Discussion

This study empirically demonstrates the complex dynamics that occur during co-colonization of a crop substrate by different aflatoxin-producing fungal species and the role of temperature on outcomes of competition. As hypothesized, temperature had a significant influence on the competition between *Aspergillus* spp., with higher temperature favoring colonization of maize kernels by the S-type species, *A. aflatoxiformans* and LAF. In contrast, the relative colonizing ability of *A. parasiticus* was greater at the lower temperature (25°C). In most cases, aflatoxin production by both individual and paired isolates increased at the higher temperature as did the relative proportion of B vs. G aflatoxins. Competition suppressed sporulation by L-type isolates of *A. flavus* and *A. parasiticus*, but the response of the S-type fungi was variable. With a few exceptions, the results of competition during colonization was predictive of the relative sporulation of co-inoculated species. Overall, results indicate that temperature will influence both the structure of fungal communities associated with a crop and the quantity and types of aflatoxins produced.

The ability of a pathogen to colonize and effectively exploit a host substrate is crucial for its growth and reproduction. In addition, colonization is of special concern when it comes to aflatoxin contamination events. For example, it has been previously demonstrated that *A. flavus* individuals that are highly competitive during colonization have the greatest influence on aflatoxin content within infected host tissues (Cotty, 1989; Mehl and Cotty, 2010). In this study, differences in relative kernel colonization of *Aspergillus* spp. were observed with a shift in temperature from 25 to 30°C. At 30°C the S-type species were more competitive than L-types *A. flavus* and *A. parasiticus*, suggesting that at higher temperatures, S-type species dominate somatic growth and potentially have greater access to nutrient resources essential for growth and reproduction. Furthermore, by dominating mycelial growth within the substrate, the S-type fungi could be the major contributors of aflatoxins in the crop when both species are present in the same field. It has been repeatedly observed that S-type fungi are the major contributors of aflatoxin contamination even if they comprise a relatively small proportion of the overall fungal community associated with the crop (Cotty, 1997; Cardwell and Cotty, 2002; Atehnkeng et al., 2008a). Thus, not only do these species have high aflatoxin-producing potential, their increased competitive ability during colonization at higher temperatures may favor dominance of S-type species in the crop-infecting fungal community.

The reduction in sporulation by the L-type fungi *A. flavus* and *A. parasiticus* and the increase or no change in sporulation by the S-type fungi during competition suggests that S-type species have the ability to modulate the behavior of other species. The S-type species may be enhancing their reproductive success over those species that produce copious quantities of conidia in the absence of competition by maintaining or increasing their sporulation while suppressing sporulation by L-type fungi. This ability to produce more conidia during competition thus enhances ability to colonize new substrates and to increase one's frequency in the environment (Jaime-Garcia and Cotty, 2004; Mehl and Cotty, 2010; Mehl et al., 2012). By maintaining or increasing sporulation while suppressing sporulation by L-type fungi, the S-type species may gain a dispersal advantage over L-type species that produce copious quantities of conidia in the absence of competition. Sporulation behavior of the S-type fungi during competition with the L-type fungi may reflect differences in growth strategies between the two sclerotial types, with each type shifting resources from somatic growth to production of conidia under different conditions resulting in increased dispersal and of each sclerotial type under different conditions (Mehl and Cotty, 2010). Although we did not quantify sclerotia in this study, Garber and Cotty (1997) reported a reduction in sclerotial formation when *A. flavus* L-type and S-type competed. In addition, Garber and Cotty (1997) assumed total spores produced during competition between *A. flavus* L-type and S-type belonged primarily to the *A. flavus* L-type. However, in the current study we discovered that S-type fungi produce greater quantities of conidia than expected during competition with an L-type *A. flavus* isolate based on sporulation in the absence of competition. In addition, since the S-type fungi were better colonizers than the L-type fungi at the higher temperature, competitive exclusion and greater access to host resources would provide S-type species with nutrients necessary for growth and dispersal (Cotty and Bayman, 1993; Lee and Magan, 2000; Mehl and Cotty, 2010; Hruska et al., 2014). It is also possible that the reduction in sporulation by the L-type species during competition resulted from production of secondary metabolites by S-type species that inhibited sporulation by the competitors during competition for resources (Losada et al., 2009; Mehl and Cotty, 2013b). This is supported by co-cultivation competition experiments that found novel secondary metabolites may be produced through activation of silent gene clusters in *Aspergillus* species (Brakhage and Schroeckh, 2011).

The apparent difference in growth strategy observed for the S-type species compared to the L-type suggests that niche partitioning exists between these fungi, allowing for their coexistence in the environment (Bayman and Cotty, 1991; Mehl and Cotty, 2010; Ohkura et al., 2018). The change in strategy manifested by the interacting species at different temperatures suggests that temperature may also influence niche partitioning (Fitt et al., 2006), with warmer temperatures driving the selection of highly toxigenic S-type fungi (Bock et al., 2004; Jaime-Garcia and Cotty, 2010). Thus, over time, consistently warmer temperatures could shift the *Aspergillus* community structure making S-type fungi more prevalent in the environment. Consistent with this observation, other field studies (Nesci and Etcheverry, 2002; Barros et al., 2005; Giorni et al., 2007; Atehnkeng et al., 2008a; Donner et al., 2009; Probst et al., 2010; Diedhiou et al., 2011; Ortega-Beltran et al., 2015; Kachapulula et al., 2017; Agbetiameh et al., 2018; Sserumaga et al., 2020) have reported high frequencies of S-type species across warm regions. In regions where *Aspergillus* species co-occur, changes in weather patterns such as shifts to prolonged hot and dry seasons as a result of climate change might favor increases in the frequency of highly aflatoxigenic S-type fungi associated with crops, thus increasing the frequency and severity of aflatoxin contamination.

Outcomes of competition and the quantity and types of aflatoxins produced by different *Aspergillus* species greatly impact the extent to which crops are contaminated with aflatoxins and thus have the potential to affect both animal and human health. Although all the isolates used in this study produce high concentrations of aflatoxins, aflatoxin concentrations in co-inoculation treatments were sometimes greater or less than expected based on outcomes of competition and aflatoxin production of individual isolates. For example, although only a single isolate was evaluated for each species in this study, *A. aflatoxiformans* in competition with the L-type species *A. flavus* and *A. parasiticus* resulted in greater than expected aflatoxin while LAF in competition with these species resulted in less aflatoxin than expected. Thus, interactions between *Aspergillus* species can be synergistic or antagonistic in terms of aflatoxin production, with consequences on overall aflatoxin production potential of the fungal community dependent on which species are present. Total aflatoxins produced by both the paired and individually cultivated isolates were high at both temperatures evaluated; this was expected since other studies have found that temperatures ranging from 25 to 35°C are favorable for aflatoxin production (Ogundero, 1987; Schmidt-Heydt et al., 2009; Singh et al., 2020). Except for *A. flavus* alone and *A. flavus* with LAF, paired and individual species produced more than twice as much aflatoxin with an increase in temperature from 25 to 30°C. In addition, the proportion of total aflatoxins comprised of B aflatoxins increased with increasing temperature in most of the treatments that included species that produce both B and G aflatoxins. The change in proportion of B aflatoxins can be explained by differences in expression ratios of *aflR* and *aflS* genes. Lower temperatures have been shown to favor expression of the *aflS* gene, which results in increased G aflatoxin production, while higher temperatures are associated with an increase in *aflR* gene expression that is associated with B aflatoxin production (Schmidt-Heydt et al., 2010). Thus, higher temperatures may increase the severity and incidence of aflatoxin contamination (Probst et al., 2010) as well as an increasing concentration of the more carcinogenic B aflatoxins in crops compared to G aflatoxins.

As demonstrated in the current study, the complex interplay between competing *Aspergillus* species and temperature has important implications for both incidence and severity of aflatoxin contamination in crops. Despite this, little attention has been paid when assessing aflatoxin mitigation methods in specific regions that vary in both temperature during the growing season and composition of aflatoxigenic species associated with crops. Thus, there is a need for future studies to address the impacts of other abiotic and biotic factors (e.g., host, other competing microorganisms, moisture, pH) on the degree of aflatoxin contamination on crops. In addition, since this study only examined the behavior of a single isolate of each species, future studies should compare multiple isolates within a species to ascertain if their behavior during competition is species or isolate specific (or both). Results from this study may also have implications on aflatoxin management strategies that aim at modulating aflatoxigenic fungal communities, such as the use of non-aflatoxigenic (atoxigenic) *A. flavus* as a biocontrol agent (Cotty, 1994). During the initial stages of selection of atoxigenic *A. flavus* for development of aflatoxin biocontrol products, candidate atoxigenic isolates are typically co-inoculated with an aflatoxigenic *A. flavus* isolate in a laboratory competition assay at 31°C (Atehnkeng et al., 2008b; Mauro et al., 2015; Agbetiameh et al., 2019). However, this study suggests that competition experiments for biocontrol strain selection should be conducted against multiple aflatoxigenic species at multiple temperatures in order to determine which atoxigenic *A. flavus* will be the most effective at displacing and reducing aflatoxin production by a variety of aflatoxin-producing species across a range of environments. In addition, the implementation of biocontrol strategies should take into consideration seasonal changes in temperature across regions and the effects of climate change, both of which are capable of shifting *Aspergillus* population structure, especially when high temperature events favor highly toxigenic S-type fungi. With increasing global temperatures, the performance of biological control strains should be periodically assessed with respect to their effectiveness against a shifting *Aspergillus* community structure.

## Data Availability Statement

The datasets generated for this study are available upon request to the corresponding author.

## Author Contributions

CC and PC contributed to conception and design of the study. CC performed the experiments, HM helped with data analysis CC wrote first draft of the manuscript. HM, KC, RB, JA, MO, and PC provided supervision. All authors contributed to manuscript revision and read and approved the submitted version.

## Author Disclaimer

Mention of trade names or commercial products in this publication is solely to provide specific information and does not imply recommendation or endorsement by the U.S. Departure of Agriculture. The U.S. Department of Agriculture is an Equal Opportunity Employer.

## Conflict of Interest

The authors declare that the research was conducted in the absence of any commercial or financial relationships that could be construed as a potential conflict of interest.

## Publisher's Note

All claims expressed in this article are solely those of the authors and do not necessarily represent those of their affiliated organizations, or those of the publisher, the editors and the reviewers. Any product that may be evaluated in this article, or claim that may be made by its manufacturer, is not guaranteed or endorsed by the publisher.

## References

[B1] Agbetiameh D. Ortega-Beltran A. Awuah R. T. Atehnkeng J. Cotty P. J. Bandyopadhyay R. (2018). Prevalence of aflatoxin contamination in maize and groundnut in Ghana: population structure, distribution, and toxigenicity of the causal agents. Plant Dis. 102, 764–772. 10.1094/PDIS-05-17-0749-RE 30673407 PMC7779968

[B2] Agbetiameh D. Ortega-Beltran A. Awuah R. T. Atehnkeng J. Islam M. S. Callicott K. A.. (2019). Potential of atoxigenic *Aspergillus flavus* vegetative compatibility groups associated with maize and groundnut in Ghana as biocontrol agents for aflatoxin management. Front. Microbiol. 10:2069. 10.3389/fmicb.2019.02069 31555251 PMC6743268

[B3] Atehnkeng J. Ojambo P. S. Ikotun T. Sikora R. A. Cotty P. J. Bandyopadhyay R. (2008b). Evaluation of atoxigenic isolates of *Aspergillus flavus* as potential biocontrol agents for aflatoxin in maize. Food Addit. Contam. 25, 1264–1271. 10.1080/02652030802112635 18608502

[B4] Atehnkeng J. Ojiambo P. S. Donner M. Ikotun T. Sikora R. A. Cotty P. J.. (2008a). Distribution and toxigenicity of *Aspergillus* species isolated from maize kernels from three agro-ecological zones in Nigeria. Int. J. Food Microbiol. 122, 74–84. 10.1016/j.ijfoodmicro.2007.11.062 18180068

[B5] Barros G. Torres A. Chulze S. (2005). *Aspergillus flavus* population isolated from soil of Argentina's peanut-growing region: sclerotia production and toxigenic profile. J. Sci. Food Agric. 85, 2349–2353. 10.1002/jsfa.2257

[B6] Bayman P. Cotty P. J. (1991). Vegetative compatibility and genetic diversity in the *Aspergillus flavus* population of a single field. Can. J. Bot. 69, 1707–1711. 10.1139/b91-216

[B7] Bhatnagar D. Cary J. W. Ehrlich K. Yu J. Cleveland T. E. (2006). Understanding the genetics of regulation of aflatoxin production and *Aspergillus flavus* development. Mycopathologia 162:155. 10.1007/s11046-006-0050-9 16944283

[B8] Bock C. H. Cotty P. J. (1999). Wheat seed colonized with atoxigenic *Aspergillus flavus*: characterization and production of a biopesticide for aflatoxin control. Biocontrol Sci. Tech. 9, 529–543. 10.1080/09583159929497

[B9] Bock C. H. Mackey B. Cotty P. J. (2004). Population dynamics of *Aspergillus flavus* in the air of an intensively cultivated region of south-west Arizona. Plant Pathol. 53, 422–433. 10.1111/j.0032-0862.2004.01015.x

[B10] Brakhage A. A. Schroeckh V. (2011). Fungal secondary metabolites–strategies to activate silent gene clusters. Fungal Genet. Biol. 48, 15–22. 10.1016/j.fgb.2010.04.004 20433937

[B11] Callicott K. A. Cotty P. J. (2015). Method for monitoring deletions in the aflatoxin biosynthesis gene cluster of *Aspergillus flavus* with multiplex PCR. Lett. Appl. Microbiol. 60, 60–65. 10.1111/lam.12337 25274127

[B12] Cardwell K. F. Cotty P. J. (2002). Distribution of *Aspergillus* section *Flavi* among field soils from the four agroecological zones of the Republic of Benin, West Africa. Plant Dis. 86, 434–439. 10.1094/PDIS.2002.86.4.434 30818721

[B13] Cotty P. J. (1989). Virulence and cultural characteristics of two *Aspergillus flavus* strains pathogenic on cotton. Phytopathology 79, 808–814. 10.1094/Phyto-79-808

[B14] Cotty P. J. (1994). Influence of field application of an atoxigenic strain of *Aspergillus flavus* on the populations of *A. flavus* infecting cotton bolls and on the aflatoxin content of cottonseed. Phytopathology 84, 1270–1277. 10.1094/Phyto-84-1270

[B15] Cotty P. J. (1997). Aflatoxin-producing potential of communities of *Aspergillus* section *Flavi* from cotton producing areas in the United States. Mycol. Res. 101, 698–704. 10.1017/S0953756296003139

[B16] Cotty P. J. Bayman P. (1993). Competitive exclusion of a toxigenic strain of *Aspergillus flavus* by an atoxigenic strain. Phytopathology 83, 1283–1287. 10.1094/Phyto-83-1283

[B17] Cotty P. J. Bayman P. Egel D. S. Elias K. S. (1994). Agriculture, aflatoxins and *Aspergillus,* in *The Genus Aspergillus* (Boston, MA: Springer), 1–27. 10.1007/978-1-4899-0981-7_1

[B18] Cotty P. J. Cardwell K. F. (1999). Divergence of West African and North American Communities of *Aspergillus* Section *Flavi*. Appl. Environ. Microbiol. 65, 2264–2266. 10.1128/AEM.65.5.2264-2266.1999 10224034 PMC91331

[B19] Cotty P. J. Jaime-Garcia R. (2007). Influences of climate on aflatoxin producing fungi and aflatoxin contamination. Int. J. Food Microbiol. 119, 109–115. 10.1016/j.ijfoodmicro.2007.07.060 17881074

[B20] Cotty P. J. Probst C. Jaime-Garcia R. (2008). Etiology and management of aflatoxin contamination, in Mycotoxins: Detection Methods, Management, Public Health and Agricultural Trade, eds J. F. Leslie, R. Bandyopadhyay, and A. Visconti (Wallingworth: CABI), 287–299. 10.1079/9781845930820.0287

[B21] Delmotte N. Knief C. Chaffron S. Innerebner G. Roschitzki B. Schlapbach R.. (2009). Community proteogenomics reveals insights into the physiology of phyllosphere bacteria. Proc. Natl. Acad. Sci. U. S. A. 106, 16428–16433. 10.1073/pnas.0905240106 19805315 PMC2738620

[B22] Diedhiou P. M. Bandyopadhyay R. Atehnkeng J. Ojiambo P. S. (2011). *Aspergillus* colonization and aflatoxin contamination of maize and sesame kernels in two agro-ecological zones in Senegal. J. Phytopathol. 159, 268–275. 10.1111/j.1439-0434.2010.01761.x

[B23] Diener U. L. Cole R. J. Sanders T. H. Payne G. A. Lee L. S. Klich M. A. (1987). Epidemiology of aflatoxin formation by *Aspergillus flavus*. Annu. Rev. Phytopathol. 25, 249–270. 10.1146/annurev.py.25.090187.001341

[B24] Donner M. Atehnkeng J. Sikora R. A. Bandyopadhyay R. Cotty P. J. (2009). Distribution of *Aspergillus* section *Flavi* in soils of maize fields in three agroecological zones of Nigeria. Soil Biol. Biochem. 41, 37–44. 10.1016/j.soilbio.2008.09.013

[B25] Edgar R. C. (2004). MUSCLE: multiple sequence alignment with high accuracy and high throughput. Nucleic Acids Res. 32, 1792–1797. 10.1093/nar/gkh340 15034147 PMC390337

[B26] Fitt B. D. Huang Y. J. van den Bosch F. West J. S. (2006). Coexistence of related pathogen species on arable crops in space and time. Annu. Rev. Phytopathol. 44, 163–182. 10.1146/annurev.phyto.44.070505.143417 16602949

[B27] Frisvad J. C. Hubka V. Ezekiel C. N. Hong S. B. Novákov,á A. Chen A. J.. (2019). Taxonomy of *Aspergillus* section *Flavi* and their production of aflatoxins, ochratoxins and other mycotoxins. Stud. Mycol. 93, 1–63. 10.1016/j.simyco.2018.06.001 30108412 PMC6080641

[B28] Garber R. K. Cotty P. J. (1997). Formation of sclerotia and aflatoxins in developing cotton bolls infected by the S strain of *Aspergillus flavus* and potential for biocontrol with an atoxigenic strain. Phytopathology 87, 940–945. 10.1094/PHYTO.1997.87.9.940 18945065

[B29] Giorni P. Magan N. Pietri A. Bertuzzi T. Battilani P. (2007). Studies on *Aspergillus* section *Flavi* isolated from maize in northern Italy. Int. J. Food Microbiol. 113, 330–338. 10.1016/j.ijfoodmicro.2006.09.007 17084935

[B30] Gong Y. Hounsa A. Egal S. Turner P. C. Sutcliffe A. E. Hall A. J.. (2004). Postweaning exposure to aflatoxin results in impaired child growth: a longitudinal study in Benin, West Africa. Environ. Health Perspect. 112, 1334–1338. 10.1289/ehp.6954 15345349 PMC1247526

[B31] Hernandez-Martinez R. Navarro-Blasco I. (2010). Aflatoxin levels and exposure assessment of Spanish infant cereals. Food Addit. Contam. 3, 275–288. 10.1080/19393210.2010.531402 24779628

[B32] Hiscox J. Clarkson G. Savoury M. Powell G. Savva I. Lloyd M.. (2016). Effects of pre-colonisation and temperature on interspecific fungal interactions in wood. Fungal Ecol. 21, 32–42. 10.1016/j.funeco.2016.01.011

[B33] Horn B. W. (2003). Ecology and population biology of aflatoxigenic fungi in soil. J. Toxicol. Toxin. Rev. 22, 351–379. 10.1081/TXR-120024098

[B34] Horn B. W. (2005). Colonization of wounded peanut seeds by soil fungi: selectivity for species from *Aspergillus* section *Flavi*. Mycologia 97, 202–217. 10.3852/mycologia.97.1.202 16389972

[B35] Hruska Z. Rajasekaran K. Yao H. Kinkaid R. Darlington D. Brown R. L.. (2014). Co-inoculation of aflatoxigenic and non-aflatoxigenic strains of *Aspergillus flavus* to study fungal invasion, colonization, and competition in maize kernels. Front. Microbiol. 5, 122. 10.3389/fmicb.2014.00122 PMC3973917 24734028

[B36] Jaime-Garcia R. Cotty P. J. (2004). *Aspergillus flavus* in soils and corncobs in south Texas: implications for management of aflatoxins in corn-cotton rotations. Plant Dis. 88, 1366–1371. 10.1094/PDIS.2004.88.12.1366 30795199

[B37] Jaime-Garcia R. Cotty P. J. (2010). Crop rotation and soil temperature influence the community structure of *Aspergillus flavus* in soil. Soil Biol. Biochem. 42, 1842–1847. 10.1016/j.soilbio.2010.06.025

[B38] Kachapulula P. W. Akello J. Bandyopadhyay R. Cotty P. J. (2017). *Aspergillus* section *Flavi* community structure in Zambia influences aflatoxin contamination of maize and groundnut. Int. J. Food Microbiol. 261, 49–56. 10.1016/j.ijfoodmicro.2017.08.014 28915412 PMC5644832

[B39] Klich M. A. (2007). *Aspergillus flavus*: the major producer of aflatoxin. Mol. Plant Pathol. 8, 713–722. 10.1111/j.1364-3703.2007.00436.x 20507532

[B40] Lee H. B. Magan N. (2000). Impact of environment and interspecific interactions between spoilage fungi and *Aspergillus ochraceus* on growth and ochratoxin production in maize grain. Int. J. Food Microbiol. 61, 11–16. 10.1016/S0168-1605(00)00385-8 11028955

[B41] Lindow S. E. Brandl M. T. (2003). Microbiology of the phyllosphere. Appl. Environ. Microbiol. 69, 1875–1883. 10.1128/AEM.69.4.1875-1883.2003 12676659 PMC154815

[B42] Liu Y. Wu F. (2010). Global burden of aflatoxin-induced hepatocellular carcinoma: a risk assessment. Environ. Health Perspect. 118, 818–824. 10.1289/ehp.0901388 20172840 PMC2898859

[B43] Losada L. Ajayi O. Frisvad J. C. Yu J. Nierman W. C. (2009). Effect of competition on the production and activity of secondary metabolites in *Aspergillus* species. Med. Mycol. 47, 88–96. 10.1080/13693780802409542 19255906

[B44] Manabe M. Tsuruta O. Goto T. Matsuura S. (1978). Study on distribution of mycotoxin-producing fungi, 4: Mycotoxin-producing ability of *Aspergillus* strains inhabited in Southeast Asia. *Rep. Natl. Food. Res. Inst*. 33, 49–56.

[B45] Mauro A. Battilani P. Cotty P. J. (2015). Atoxigenic *Aspergillus flavus* endemic to Italy for biocontrol of aflatoxins in maize. BioControl 60, 125–134. 10.1007/s10526-014-9624-5

[B46] Mehl H. L. Cotty P. J. (2010). Variation in competitive ability among isolates of *Aspergillus flavus* from different vegetative compatibility groups during maize infection. Phytopathology 100, 150–159. 10.1094/PHYTO-100-2-0150 20055649

[B47] Mehl H. L. Cotty P. J. (2013a) Influence of plant host species on intraspecific competition during infection by Aspergillus flavus. Plant Pathol. 62, 1310–1318.

[B48] Mehl H. L. Cotty P. J. (2013b). Nutrient environments influence competition among *Aspergillus flavus* genotypes. Appl. Environ. Microbiol. 79, 1473–1480. 10.1128/AEM.02970-12 23263958 PMC3591962

[B49] Mehl H. L. Jaime R. Callicott K. A. Probst C. Garber N. P. Ortega-Beltran A.. (2012). *Aspergillus flavus* diversity on crops and in the environment can be exploited to reduce aflatoxin exposure and improve health. Ann. N. Y. Acad. Sci. 1273, 7–17. 10.1111/j.1749-6632.2012.06800.x 23230832

[B50] Mitchell N. J. Bowers E. Hurburgh C. Wu F. (2016). Potential economic losses to the US corn industry from aflatoxin contamination. Food Addit. Contam. 33, 540–550. 10.1080/19440049.2016.1138545 26807606 PMC4815912

[B51] Nesci A. Etcheverry M. (2002). *Aspergillus* section *Flavi* populations from field maize in Argentina. Lett. Appl. Microbiol. 34, 343–348. 10.1046/j.1472-765X.2002.01094.x 11967056

[B52] O'Brian G. R. Georgianna D. R. Wilkinson J. R. Yu J. Abbas H. K. Bhatnagar D.. (2007). The effect of elevated temperature on gene transcription and aflatoxin biosynthesis. Mycologia 99, 232–239. 10.1080/15572536.2007.11832583 17682776

[B53] Ogundero V. W. (1987). Temperature and aflatoxin production by *Aspergillus flavus* and *A. parasiticus* strains from Nigerian groundnuts. J. Basic Microbiol. 27, 511–514. 10.1002/jobm.3620270910 3136240

[B54] Ohkura M. Cotty P. J. Orbach M. J. (2018). Comparative genomics of *Aspergillus flavus* S and L morphotypes yield insights into niche adaptation. G3 Genes Genom. Genet. 8, 3915–3930. 10.1534/g3.118.200553 30361280 PMC6288828

[B55] Ortega-Beltran A. Jaime R. Cotty P. J. (2015). Aflatoxin-producing fungi in maize field soils from sea level to over 2000 masl: a three year study in Sonora, Mexico. Fungal Biol. 119, 191–200. 10.1016/j.funbio.2014.12.006 25813508

[B56] Payne G. A. Cassel D. K. Adkins C. R. (1985). Reduction of aflatoxin levels in maize due to irrigation and tillage. Phytopathology 75, 1283–1283.

[B57] Perrone G. Gallo A. Logrieco A. F. (2014). Biodiversity of *Aspergillus* section *Flavi* in Europe in relation to the management of aflatoxin risk. Front. Microbiol. 5:377. 10.3389/fmicb.2014.00377 25101075 PMC4104701

[B58] Pildain M. B. Frisvad J. C. Vaamonde G. Cabral D. Varga J. Samson R. A. (2008). Two novel aflatoxin-producing *Aspergillus* species from Argentinean peanuts. Int. J. Syst. Evol. Microbiol. 58, 725–735. 10.1099/ijs.0.65123-0 18319485

[B59] Pildain M. B. Vaamonde G. Cabral D. (2004). Analysis of population structure of *Aspergillus flavus* from peanut based on vegetative compatibility, geographic origin, mycotoxin and sclerotia production. Int. J. Food Microbiol. 93, 31–40. 10.1016/j.ijfoodmicro.2003.10.007 15135580

[B60] Pitt J. I. Miscamble B. F. (1995). Water relations of *Aspergillus flavus* and closely related species. J. Food Protect. 58, 86–90. 10.4315/0362-028X-58.1.86 31121770

[B61] Pons W. A. Jr. Robertson J. A. Goldblatt L. A. (1969). Collaborative study on the determination of aflatoxins in cottonseed products. J. Assoc. Off. Anal. Chem. 51, 61–72. 10.1093/jaoac/52.1.61

[B62] Probst C. Callicott K. A. Cotty P. J. (2012). Deadly strains of Kenyan *Aspergillus* are distinct from other aflatoxin producers. Eur. J. Plant Pathol. 132, 419–429. 10.1007/s10658-011-9887-y

[B63] Probst C. Cotty P. J. (2012). Relationships between *in vivo* and *in vitro* aflatoxin production: reliable prediction of fungal ability to contaminate maize with aflatoxins. Fungal Biol. 116, 503–510. 10.1016/j.funbio.2012.02.001 22483048

[B64] Probst C. Njapau H. Cotty P. J. (2007). Outbreak of an acute aflatoxicosis in Kenya in 2004: identification of the causal agent. Appl. Environ. Microbiol. 73, 2762–2764. 10.1128/AEM.02370-06 17308181 PMC1855601

[B65] Probst C. Schulthess F. Cotty P. J. (2010). Impact of *Aspergillus* section *Flavi* community structure on the development of lethal levels of aflatoxins in Kenyan maize (*Zea mays*). *J. Appl. Microbiol*. 108, 600–610. 10.1111/j.1365-2672.2009.04458.x 19674186

[B66] Rambo G. W. Tuite J. Crane P. (1974). Preharvest inoculation and infection of dent corn ears with *Aspergillus flavus* and *Aspergillus parasiticus*. Phytopathology 64, 797–800. 10.1094/Phyto-64-797

[B67] Scheidegger K. A. Payne G. A. (2003). Unlocking the secrets behind secondary metabolism: a review of *Aspergillus flavus* from pathogenicity to functional genomics. J. Toxicol. Toxin. Rev. 22, 423–459. 10.1081/TXR-120024100

[B68] Schmidt-Heydt M. Abdel-Hadi A. Magan N. Geisen R. (2009). Complex regulation of the aflatoxin biosynthesis gene cluster of *Aspergillus flavus* in relation to various combinations of water activity and temperature. Int. J. Food Microbiol. 135, 231–237. 10.1016/j.ijfoodmicro.2009.07.026 19699547

[B69] Schmidt-Heydt M. Rufer C. E. Abdel-Hadi A. Magan N. Geisen R. (2010). The production of aflatoxin B_1_ or G_1_ by *Aspergillus parasiticus* at various combinations of temperature and water activity is related to the ratio of *aflS* to *aflR* expression. Mycotoxin Res. 26, 241–246. 10.1007/s12550-010-0062-7 23605486

[B70] Shearer J. F. Sweets L. E. Baker N. K. Tiffany L. H. (1992). A study of *Aspergillus flavus/parasiticus* in Iowa crop fields: 1988-1990. Plant Dis. 76, 19–22. 10.1094/PD-76-0019

[B71] Singh P. Callicott K. A. Orbach M. J. Cotty P. J. (2020). Molecular analysis of S-morphology aflatoxin producers from the United States reveals previously unknown diversity and two new taxa. Front. Microbiol. 11:1236. 10.3389/fmicb.2020.01236 32625180 PMC7315800

[B72] Singh P. Cotty P. J. (2019). Characterization of Aspergilli from dried red chilies (*Capsicum* spp.): Insights into the etiology of aflatoxin contamination. Int. J. Food Microbiol. 289, 145–153. 10.1016/j.ijfoodmicro.2018.08.025 30243147

[B73] Singh P. Orbach M. J. Cotty P. J. (2018). *Aspergillus texensis*: a novel aflatoxin producer with S morphology from the United States. Toxins 10:513. 10.3390/toxins10120513 30513994 PMC6316697

[B74] Sserumaga J. P. Ortega-Beltran A. Wagacha J. M. Mutegi C. K. Bandyopadhyay R. (2020). Aflatoxin-producing fungi associated with pre-harvest maize contamination in Uganda. Int. J. Food Microbiol. 313:108376. 10.1016/j.ijfoodmicro.2019.108376 31731141

[B75] Sweany R. R. Damann K. E. Jr Kaller M. D. (2011). Comparison of soil and corn kernel *Aspergillus flavus* populations: evidence for niche specialization. Phytopathology 101,952–959. 10.1094/PHYTO-09-10-0243 21405994

[B76] Williams J. H. Phillips T. D. Jolly P. E. Stiles J. K. Jolly C. M. Aggarwal D. (2004). Human aflatoxicosis in developing countries: a review of toxicology, exposure, potential health consequences, and interventions. Am. J. Clin. Nutr. 80, 1106–1122. 10.1093/ajcn/80.5.1106 15531656

[B77] Wu F. (2015). Global impacts of aflatoxin in maize: trade and human health. World Mycotoxin J. 8, 137–142. 10.3920/WMJ2014.1737

